# Bio-Template Synthesis of V_2_O_3_@Carbonized Dictyophora Composites for Advanced Aqueous Zinc-Ion Batteries

**DOI:** 10.3390/molecules28052147

**Published:** 2023-02-24

**Authors:** Wei Zhou, Guilin Zeng, Haotian Jin, Shaohua Jiang, Minjie Huang, Chunmei Zhang, Han Chen

**Affiliations:** 1 College of Materials and Advanced Manufacturing, Hunan University of Technology, Zhuzhou 412007, China; 2 Jiangsu Co-Innovation Center of Efficient Processing and Utilization of Forest Resources, International Innovation Center for Forest Chemicals and Materials, College of Materials Science and Engineering, Nanjing Forestry University, Nanjing 210037, China; 3 Hunan Key Laboratory of Applied Environmental Photocatalysis, Changsha University, Changsha 410022, China; 4 Institute of Materials Science and Devices, School of Materials Science and Engineering, Suzhou University of Science and Technology, Suzhou 215009, China

**Keywords:** V_2_O_3_@CD, aqueous zinc ion battery, dictyophora, cathode materials, long cycle, capacity retention

## Abstract

In terms of new-generation energy-storing devices, aqueous zinc-ion batteries (AZIBs) are becoming the prime candidates because of their inexpensive nature, inherent safety, environmental benignity and abundant resources. Nevertheless, due to a restrained selection of cathodes, AZIBs often perform unsatisfactorily under long-life cycling and high-rate conditions. Consequently, we propose a facile evaporation-induced self-assembly technique for preparing V_2_O_3_@carbonized dictyophora (V_2_O_3_@CD) composites, utilizing economical and easily available biomass dictyophora as carbon sources and NH_4_VO_3_ as metal sources. When assembled in AZIBs, the V_2_O_3_@CD exhibits a high initial discharge capacity of 281.9 mAh g^−1^ at 50 mA g^−1^. The discharge capacity is still up to 151.9 mAh g^−1^ after 1000 cycles at 1 A g^−1^, showing excellent long-cycle durability. The extraordinary high electrochemical effectiveness of V_2_O_3_@CD could be mainly attributed to the formation of porous carbonized dictyophora frame. The formed porous carbon skeleton can ensure efficient electron transport and prevent V_2_O_3_ from losing electrical contact due to volume changes caused by Zn^2+^ intercalation/deintercalation. The strategy of metal-oxide-filled carbonized biomass material may provide insights into developing high-performance AZIBs and other potential energy storage devices, with a wide application range.

## 1. Introduction

Lithium-ion batteries (LIBs), with the advantages of mature preparation technology and high energy density, are viewed as the most alluring candidates for advanced portable and automotive electrical energy storage systems [1,2,3,4]. Nonetheless, the deficiency in lithium assets and unpredictable safety problems of flammable electrolytes have genuinely hindered the further development and wide application of LIBs [5,6]. Consequently, it is imperative to find alternatives to LIBs. Recently, aqueous metal-ion (Zn, Na, K, Mg, Ca, etc.) batteries have exhibited extraordinary prospects for applications in energy storage, owing to their fabulous quality of security [7]. Among them, rechargeable aqueous zinc-ion batteries (AZIBs) are considered to be one of the foremost promising energy storage devices on account of their plenteous sources, nontoxicity and high theoretical capacity (820 mAh g^−1^) [8,9,10,11].

Within the past few decades, various types of host materials, including MnO_2_, Prussian blue analogues, organic compounds and V-based materials, have been considered extensively as AZIB cathodes [8,12]. However, Zn/MnO_2_ aqueous batteries feature an underdeveloped rate capability and significant capacity fading because of the sluggish dynamics and phase transitions [13,14]. Although Prussian blue analogue cathodes presented superior rate capability and cyclability, their practical application is still enormously constrained by the problem of low specific capacity of below 100 mAh g^−1^ and producing O_2_ evolution under high working voltage [15]. For organic compound electrodes, the formed discharge products are easily dissolved in the aqueous electrolyte, leading to cycling performance degradation [16]. In contrast, Vanadium oxides, with an open-layered structure, are considered as potential candidates for AZIB cathodes, which could benefit from the multivalent transition effect of vanadium to furnish the extra losses and gains of electrons, thus resulting in high theoretical capacity (generally >300 mAh g^−1^) [17]. In addition, differences in the oxidation states and REDOX properties of vanadium lead to the formation of different types of layered vanadium oxides and their vanadate, which can be applied to temperature sensors, catalysis and various optical and electrochemical devices [18,19]. Currently, the commonly employed vanadium oxides for electrode materials include V_2_O_3_ [20], H_2_V_3_O_8_ [1], CaV_4_O_9_ [21], VO_2_ [22], V_3_O_7_∙H_2_O [23], Zn_3_V_2_O_7_(OH)_2_∙2H_2_O [24], V_6_O_13_ [2] and so on.

However, vanadium oxides generally suffer from limited electrochemical performances, including dramatic capacity decay and unsatisfactory rate capability due to surface element dissolution, inferior electric conductivity and slow reaction kinetics. Previous research has indicated that compositing with carbon-based materials [25,26] cannot only construct a conducting framework for electron transfer, thus ameliorating the rate performance, but also effectively mitigate the instability of the layered structure of vanadium oxides [5]. Pang et al. [1] reported that H_2_V_3_O_8_ nanowire/graphene materials prepared via the hydrothermal method exhibited better cycling stability and far higher current density compared to a pure H_2_V_3_O_8_ electrode. Hu et al. [27] demonstrated that uniformly encapsulating V_2_O_3_ nanoparticles in amorphous carbon nanosheets can effectively promote the penetration of the electrolyte, leading to rapid and durable potassium storage behavior in PIBs. Dai et al. [28] prepared V_2_O_5_@polyaniline cathode materials for AZIBs, exhibiting a high reversible capacity of 361 mAh g^−1^ at 0.1 A g^−1^ and a lifespan of 1000 cycles (93.8% capacity retention). Tamilselvan et al. [2] designed and fabricated a V_6_O_13_@carbon cloth with excellent electrical conductivity, exhibiting an initial specific capacity of 227 mAh g^−1^ and nearly 99% retention rate after 1000 cycles. Although the above-mentioned synthetic methods are well developed in small-batch production, they are still far from commercialization due to the complex synthetic approaches, high cost and unsustainable carbon sources [29,30,31]. Thus, seeking efficient and renewable raw materials for preparing functional carbon materials has gradually gained attention. As a carbon-rich precursor, biomass has been widely studied and applied in many fields due to its natural advantages of renewable and abundant resources, environmental friendliness and economy [32,33,34,35,36].

In this work, aiming to enhance the electrochemical performance of vanadium oxides as AZIB cathodes, V_2_O_3_@carbonized dictyophora (V_2_O_3_@CD) composites were synthesized via an evaporation-induced self-assembly technique by using NH_4_VO_3_ as metal sources and carbonized dictyophora as carbon sources. Carbonized dictyophora provides a carbon skeleton for V_2_O_3_ by retaining its unique rhombohedral structure, which not only effectively alleviates the volume expansion of the electrode, but also improves the intercalation/de-intercalation process of zinc ions. Therefore, an exceptionally high capacity of 289 mAh g^−1^ at a current density of 50 mA g^−1^, a long-life cycling stability with a capacity retention rate of 89% after 1000 cycles at 50 mA g^−1^ and an excellent rate capability (170 mAh g^−1^ at 1 A g^−1^) are achieved in the as-prepared V_2_O_3_@CD composites. The V_2_O_3_@CD cathode could offer tremendous possibilities for fast and durable zinc-ion storage.

## 2. Results

**Figure 1:**
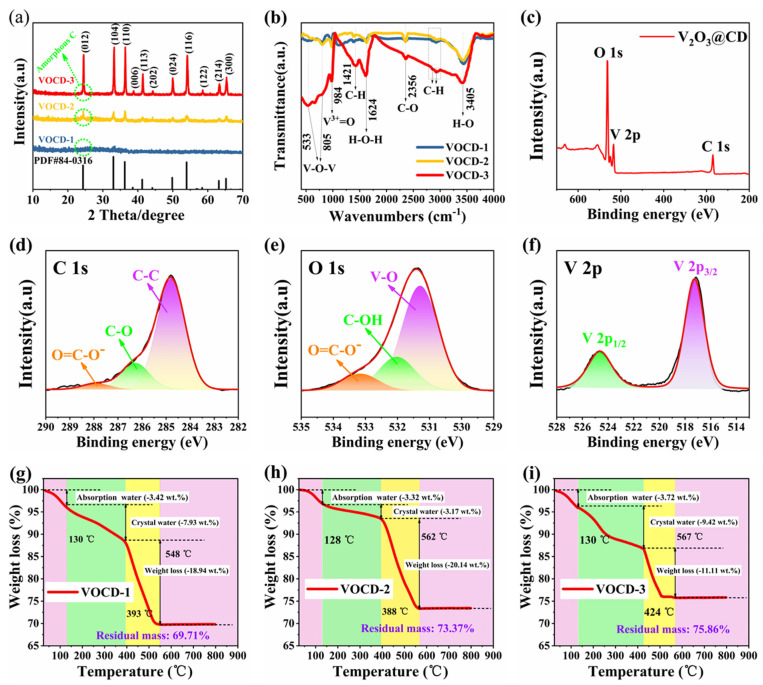
(**a**) XRD patterns, (**b**) FT-IR spectrum, (**c**) XPS survey spectra of the V_2_O_3_/CD composites, high-resolution (**d**) C 1s, (**e**) O 1s and (**f**) V 2p XPS spectrum of the V_2_O_3_/CD composites and (**g**–**i**) TGA curves of the V_2_O_3_/CD composites.

The crystalline structure of as-synthesized V_2_O_3_@CD composites was characterized by an XRD experiment. The corresponding XRD patterns (Figure 1a) manifest that the distinct narrow peaks of all products can be assigned to rhombohedral V_2_O_3_ (space group: R-3c, ICSD-01-071-0342) with the lattice parameters of a = 4.95 Å, b = 4.95 Å, c = 14.00 Å, α = 90°, β = 90° and γ = 120° [37,38]. It is worth mentioning that a low-intensity amorphous bump centered at approximately 24.5°, which can be attributed to the (002) plane of amorphous C, and the intensity of this amorphous C peak, debilitates with the diminishing of the C atom substance [27]. No other noticeable impurity phases were detected in the pattern, suggesting that V_2_O_3_ was successfully synthesized [39]. Due to the low content of V, the diffraction peaks of the VOCD-1 sample are the weakest among all the as-prepared composites. With a higher content of V, the VOCD-3 sample shows stronger diffraction peaks compared with the other two V_2_O_3_@CD composites, indicating better crystallinity, which is beneficial to the ion diffusion between the layers of the electrode.

The FT-IR spectra of V_2_O_3_@CD composites are illustrated in Figure 1b. The bending vibration of O-H and the stretching vibration of H-O-H can be observed at 1624 and 3405 cm^−1^, respectively, which is mainly because some water molecules are embedded between the layers and adsorbed on the surface of the composites [40]. The peaks at 805 and 533 cm^−1^ can be indexed to the symmetric and asymmetric stretching vibration of V-O-V [41]. Notably, the peak centered at 984 cm^−1^ is associated with the stretching vibration of the V^3+^=O, demonstrating the appearance of V_2_O_3_ [42]. The peaks at 2855 and 2932 cm^−1^ are related to the stretching vibration of C-H bonds [43], while the peak located at 1421 cm^−1^ is characteristic of bending vibration of a C-H bond [44]. The asymmetrical stretching vibration of C-O is around 2356 cm^−1^, aiming at CO_2_ adsorption on KBr, which is negligible [45]. The above FT-IR spectra demonstrate that the as-obtained composites are composed of V_2_O_3_ and amorphous carbon, which is in agreement with the XRD results.

XPS spectrum was employed to investigate the elemental valence and surface composition of V_2_O_3_/CD composites. As illustrated in Figure 1c–f, all the signal peaks can be assigned to C 1s, O 1s and V 2p in the survey spectra. The deconvoluted C 1s spectra of V_2_O_3_/CD composites revealed several components at 287.9, 286.3 and 284.8 eV, which corresponded to O-C=O^−^, C-O and C-C bond, respectively [46,47,48]. The O 1s spectrum of V_2_O_3_/CD presented three contributions, with binding energies of 533.2, 532.0 and 531.3 eV, which were associated with O=C-O^−^, C–OH and V-O, respectively [34,49]. In V 2p core-level spectrum, the two peaks located at 524.6 and 517.5 eV were caused by the spin–orbit splitting of V 2p_1/2_ and V 2p_3/2_, respectively, which is characteristic of vanadium in the +3 oxidation state [14,50]. Additionally, the energy difference between the binding energy of the O 1s and V 2p_3/2_ level (ΔE = E_O 1s_ − E V 2p_3/2_) could be employed to determine the oxidation states of the vanadium oxides. The ΔE value of as-obtained V_2_O_3_@CD in the present work is 14 eV, which is consistent with the values of V^3+^ compounds reported in the literature, thus confirming the vanadium valence in +3 oxidation states [44]. Therefore, the XPS spectrum further illustrates the formation of V_2_O_3_ in the composites.

TGA analyses were conducted to determine the mass content of each component in the V_2_O_3_@CD composites. Figure 1g–i reveal the TGA curves of three V_2_O_3_@CD composites. Obviously, three weight-loss stages during the decomposition of the composites are presented in the TGA curves. The first and second stages of weight loss appear at about 130 °C and 400 °C, which are correlated with the release of adsorptive and structural water, respectively. The third stage of weight loss occurs at 380–570 °C, which is mainly associated with the combustion of carbon. The weight loss of VOCD-1, VOCD-2 and VOCD-3 in the third stage is 18.94%, 20.14% and 11.11%, respectively. Based on the results of TGA analysis, the mass contents of biomass carbon and vanadium oxide in VOCD-1, VOCD-2 and VOCD-3 are 18.94% and 69.71%, 20.14% and 73.37%, and 11.11% and 75.86%, respectively.

**Figure 2:**
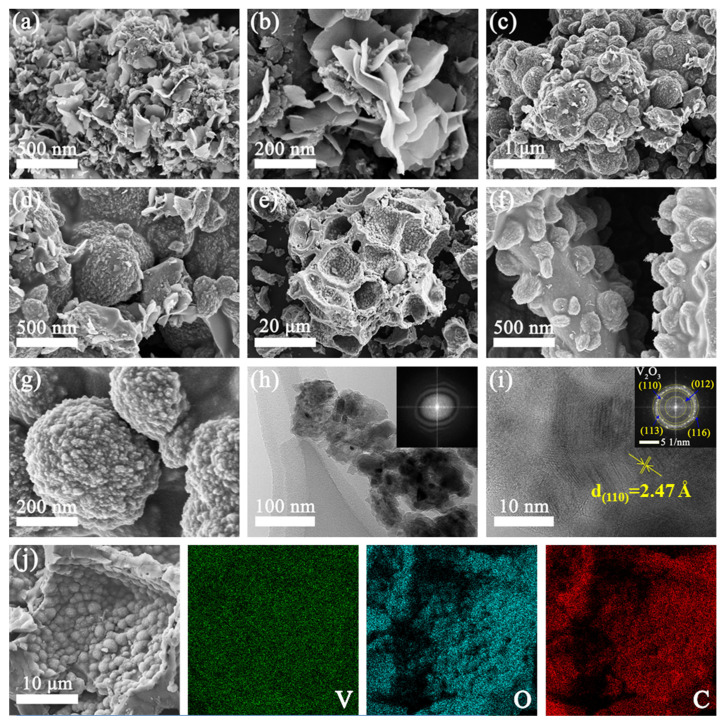
SEM images of (**a**,**b**) VOCD-1, (**c**,**d**) VOCD-2 and (**e**–**g**) VOCD-3, (**h**,**i**) HR-TEM image (inset: SEAD pattern) and (**j**) elemental mapping images of the synthesized V_2_O_3_@CD.

**Figure 3:**
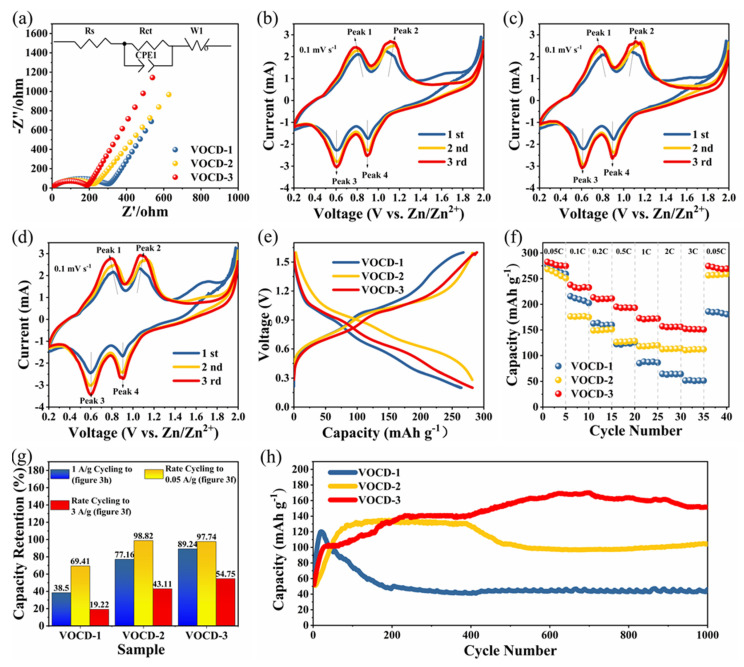
(**a**) Nyquist plots of V_2_O_3_@CD electrodes (inset: equivalent circuit simulation). (**b**–**d**) CV curves of the initial three cycles for the VOCD-2 and VOCD-3 within 0.2–2.0 V at 0.1 mV s^−1^. (**e**) Discharge–charge profiles at 0.05 A g^−1^. (**f**) Rate capability at various current densities. (**g**) Capacity retention after 1000 cycles at 1 A g^−1^, capacity retention after rate cycling to 3 A g^−1^ and rate cycling back to 0.05 A g^−1^. (**h**) Galvanostatic cycling at 1 A g^−1^.

The morphology and structure of as-obtained V_2_O_3_@CD composites were investigated using SEM, TEM and EDS, and the results are presented in Figure 2. It can be discerned that the VOCD-1 composites show an obvious petal shape (Figure 2a,b), which may be due to the low content of V_2_O_3_ in the VOCD-1 composites. As displayed in Figure 3c,d, the structure of V_2_O_3_ in the VOCD-2 composites is mainly spherical with a diameter of 0.5–1.3 μm and a small number of sheets. The VOCD-3 composites display a unique porous architecture, and the diameter of pores is 6–12 μm (Figure 2e), indicating that the incorporation of V_2_O_3_ has little effect on the formation of the natural porous network of CD. As illustrated in Figure 2f,g, the structure of V_2_O_3_ in the VOCD-3 composites is spherical, with almost no sheets, and these microspheres are evenly filled in the macropores of CD. It is noteworthy that the surfaces of the V_2_O_3_ microspheres in the VOCD-2 and VOCD-3 composites are very coarse, indicating that both have a well-developed pore structure. The diffraction rings obtained from selected-area electron diffraction (SEAD) analysis (inset of Figure 2h) indicate the polycrystalline character of the sample. The inserted SAED pattern (Figure 2i) exhibits four distinctive diffraction rings belonging to the (012), (110), (113) and (116) planes of V_2_O_3_, respectively, demonstrating the existence of V_2_O_3_ in V_2_O_3_@CD. In addition, the HR-TEM image (inset of Figure 2i) manifests a crystal structure with clear lattice fringe of 2.47 Å that can be assigned to the d-spacing of (110) planes of the rhombohedral phase of crystalline V_2_O_3_, further verifying the presence of V_2_O_3_ in V_2_O_3_@CD [51]. The corresponding element mapping images (Figure 2j) reveal the homogenous distribution of C, O and V elements in the composites, indicating that V_2_O_3_ is homogeneously distributed on the surface and within the macropores of the porous CD matrix. The porous carbon with evenly distributed V_2_O_3_ is anticipated to endow V_2_O_3_@CD composites significantly increased pathways for alleviating the volume expansion, favoring the diffusion of electrolyte and improving the transfer of the electron, thus enhancing the electrochemical storage capacity of zinc.

## 3. Discussion

To explore the electrochemical behavior of V_2_O_3_@CD electrodes, EIS tests were carried out and corresponding Nyquist plots and the equivalent circuit are gathered in Figure 3a. The curves of V_2_O_3_@CD electrodes consist of one sloped line at low frequencies, associated with Warburg impedance and one semi-circle at a high-to-medium frequency region, which could be regarded as the charge transfer resistance (R_ct_) [52]. The VOCD-3 electrode exhibits the lowest R_ct_ of 187.7 Ω compared to VOCD-1 (307.9 Ω) and VOCD-2 (220.4 Ω), demonstrating that the VOCD-3 electrode has the best electronic conductivity. Although V_2_O_3_ has higher resistance and ion resistance than CD, the R_ct_ value is also related to the pore structure and the bonding strength between CD [53]. Therefore, the R_ct_ of VOCD-3 is the lowest among the composites. The CV curves were administered to evaluate the kinetics of the electrochemical process of V_2_O_3_@CD electrodes, and the initial three cycles are illustrated in Figure 3b–d. The CV curves exhibit similar shapes, with two coupled redox peaks located at approximately 0.62/0.81 V and 0.91/1.11 V, manifesting that a multi-step reaction associated with Zn^2+^ ion (de)insertion process occurs in the V_2_O_3_@CD electrode [9,54]. In addition, VOCD-3 presents the widest redox peaks in the CV curve among the V_2_O_3_@CD composites, which is conducive to the kinetics of Zn^2+^ (de)insertion, thus exhibiting the highest specific capacity [55]. It is worth noting that with an increase in cycle times, the area in the CV curve also increases, illustrating an increase in the capacity of V_2_O_3_@CD electrodes at the early stage of the electrochemical cycle process (see Figure 3g).

The galvanostatic charge/discharge (GCD) profiles of V_2_O_3_@CD electrodes cycled at 50 mA g^−1^ between 0.2 and 1.6 V versus Zn^2+^/Zn are displayed in Figure 3e. Multiple redox pairs of voltage plateaus at 0.9/1.1 and 0.6/0.8 V can be observed on each charge and discharge curve, which can be consistent with the CV analysis. Remarkably, VOCD-3 can obtain a distinguished reversible capacity of 281.94 mAh g^−1^, which is higher than VOCD-1 of 264.47 mAh g^−1^ and VOCD-2 of 281.79 mAh g^−1^ at the initial cycle. The rate capabilities at different current densities, ranging from 0.05 C to 3 C for the V_2_O_3_@CD electrodes, are depicted in Figure 3f. In the three groups of rate capability, the specific capacity continues to decrease, regardless of the increase in current densities. VOCD-3 reveals significantly higher discharge capacities of 276.5, 231.60, 210.61, 193.60, 171.76, 155.81 and 151.38 mAh g^−1^ at 0.05, 0.1, 0.2, 0.5, 1, 2 and 3 C, respectively, compared to VOCD-1 and VOCD-2. When the current density is withdrawn to 0.05 C, VOCD-2 and VOCD-3 electrodes can recover to a high capacity, maintained at 257.33 and 270.24 mAh g^−1^, corresponding to 98.82% and 97.74% of their initial capacity, respectively, while VOCD-1 can retain only 69.41% of its initial capacity (184.86 mAh g^−1^) (Figure 3f,g). The above results demonstrate that the VOCD-3 electrode possesses the best rate performance compared with VOCD-1 and VOCD-2, which can be mainly attributed to the unique porous architectures of CD and high content of V_2_O_3_.

Figure 3h presents the long-cycle performance of V_2_O_3_@CD electrodes at 1 A g^−1^. The capacity of the three V_2_O_3_@CD composites increases gradually in the initial cycles, which is probably correlated with a gradual electrochemical activation process widely existing in vanadium-based electrodes of AZIBs [6,13,46]. Remarkably, VOCD-3 exhibits the highest capacity, up to 170.19 mAh g^−1^ after 693 cycles, among the three V_2_O_3_@CD composites. After 1000 cycles, the capacity of VOCD-3 slightly decreases to 151.89 mAh g^−1^, possessing capacity retention of 89.2%. In contrast, VOCD-1 and VOCD-2 show much lower capacities of 46.27 and 104.12 mAh g^−1^, with inferior capacity retentions of 38.5% and 77.2%, respectively, mainly due to their higher content of CD, resulting in lower reversible capacity. It has been reported that low levels of V_2_O_3_ may not take advantage of the benefits of V_2_O_3_, while high levels of V_2_O_3_ may lead to low electrical conductivity and poor CD protection [34]. The excellent electrochemical performance of VOCD-3 can be ascribed to the high content of V_2_O_3_ active and unique porous architectures in the CD matrix. The high content of V_2_O_3_ is beneficial to achieving a high reversible capacity. The porous architecture of CD not only effectively promotes the entrance of the electrolyte but can also perform as a buffering effect to assuage the huge volume change of V_2_O_3_ during the repeated (de)intercalation of Zn^2+^ from/into V_2_O_3_ process [15,34,56].

For a comparison, the electrochemical properties of V-based materials utilized as AZIB cathodes are collected in Table 1. The result shows that the V_2_O_3_/CD composites proposed in this work exhibit advantages. The excellent properties of V_2_O_3_/CD composites can probably be explained by the suitable ratio of V_2_O_3_ and CD to maximize their individual benefits.

**Figure 4:**
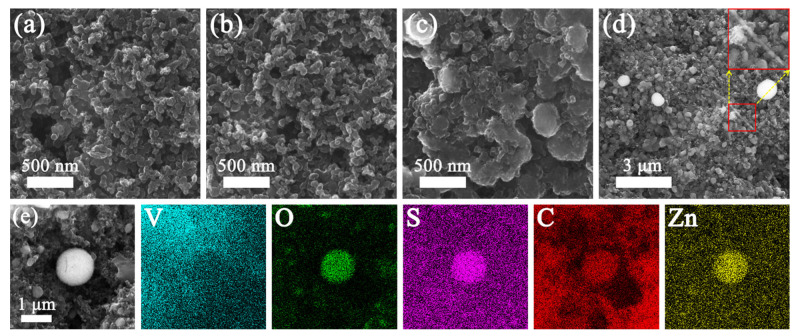
SEM images of VOCD-3 electrodes at different states of (**a**) pristine, after (**b**) 300, (**c**) 600 and (**d**) 900 cycles; (**e**) EDS mapping image of the V, O, S, C and Zn elemental distribution in the VOCD-3 cathode after 900 cycles.

To verify the structure stability, the morphology of the VOCD-3 electrode at diverse stages of pristine and after 300, 600 and 900 cycles were recorded by SEM, respectively, as shown in Figure 4a–d. In the initial state, nanoparticles are homogeneously distributed on the current collector without experiencing an agglomeration phenomenon in Figure 4a. After 300 cycles, the morphology of the VOCD-3 electrode remains almost the same as its original state, indicating good structural stability in the cycling process. After 900 cycles, apparent cracks emerged on the VOCD-3 electrode surface (Figure 4d), revealing that VOCD-3 is pulverizing during repetitive charge/discharge, thus resulting in capacity decline. From the element distribution mapping, in addition to the four elements of V, O, S and C, Zn is also detected from the electrode material, indicating that Zn^2+^ is successfully embedded in VOCD-3. In particular, no obvious dendrites are observed in the VOCD-3 electrode at the 200, 500 and 900 cycles (see Figure 4a–d). Combined with the favorable properties of Zn^2+^ storage, the protection mechanism of the porous structure can be intimated. Therefore, the as-obtained VOCD-3 composites employed as electrodes are conducive to improving the durability of the AZIBs.

## 4. Materials and Methods

### 4.1. Materials Synthesis

**Figure 5:**
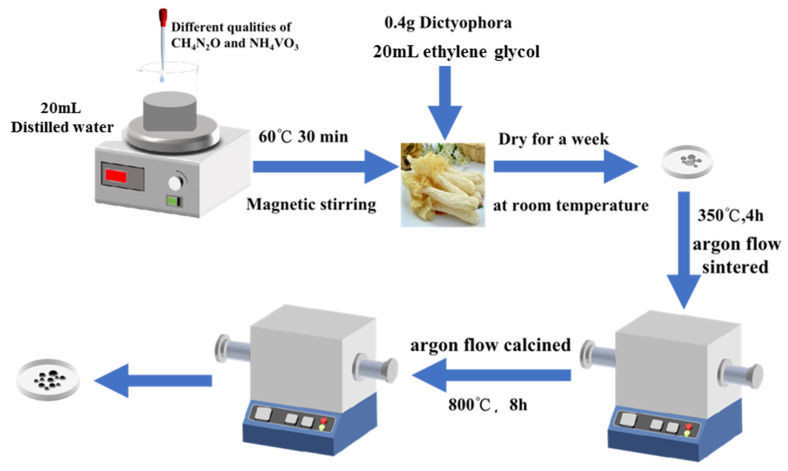
The preparation process of V_2_O_3_@CD composites.

Urea (CH_4_N_2_O, ≥99.0%, AR), ethylene glycol (C_2_H_6_O_2_, AR) and ammonium metavanadate (NH_4_VO_3_, ≥99.0%, AR) were directly utilized without any purification. The V_2_O_3_@C composites were synthesized via evaporation-induced self-assembly technique. The detailed preparation process is schematically depicted in Figure 5. Firstly, 3 g CH_4_N_2_O and 2.2 g NH_4_VO_3_ were dissolved in a beaker with 20 mL of distilled water. After continuous magnetic stirring at 60 °C for 0.5 h, 20 mL C_2_H_6_O_2_ and 0.4 g dried dictyophora were added into the above-mentioned beaker and stirred for 0.5 h to form homogeneous liquid. Then, the obtained solution was heated completely in an oven at 100 °C for 10 h. The resultant powder sample was sintered first at 350 °C for 4 h in an argon-filled tube furnace to obtain VO_2_ and carbonized dictyophora. Then, the obtained products were calcined at 800 °C for 8 h, with a heating rate of 10 °C min^−1^. The chemical reactions occurring in this process and the reaction principle can be described by Gibbs free energy (G°) as a function of temperature (T), as presented in Equation (1).

(1) $$2{\mathrm{VO}}_{2}+C\to V_{2}O_{3}+\mathrm{CO}↑,\;\Delta G{}^{\circ}\;\left(J/\mathrm{mol}\right)=95300-158.68T$$

According to the thermodynamic data, T = (t +273.5) K = 1073.5 K, ∆G° = 95300 − 158.68 T < 0, indicating that the reaction is positive. In this reaction, VO_2_ can be deoxidized to V_2_O_3_ using carbon as the reducing agent. The C acts as a reducing agent to reduce the high-valent VO_2_ to low-valent V_2_O_3_.

The final synthesized sample was named VOCD-1. For comparison, VOCD-2 and VOCD-3 were also prepared in the same way by changing the mass ratio of dictyophora to NH_4_VO_3_. The dosage of raw materials for each sample is summarized in Table 2.

### 4.2. Materials Characterization

The crystal structure of the synthesized samples was characterized by a powder Bruker D8 Advance X-ray diffractometer (XRD, Rigaku Corporation, Japan)) equipped with Cu Kα radiation (λ = 0.152 nm). The FT-IR spectra with a wavenumber range of 400–4000 cm^−1^ were recorded on an FT-IR spectrometer (Vertex-70, Bruker, Heidelberg, Germany). X-ray photoelectron spectroscopy (XPS, Kratos Analytical Ltd., Manchester, UK) analysis was conducted on a Thermo Escalab 250Xi spectrometer (Thermo Fisher Scientific, Waltham, MA, USA) employing a monochromatic Al-Kα X-ray source (Kα = 1486.6 eV). The measured binding energies were calibrated by using the containment carbon (C1s = 284.6 eV) as a reference. All XPS spectra analysis and curve fitting were carried out using PeakFit v4.12 software. The surface morphology and microstructure were determined by transmission electron microscopy equipped with Energy dispersive spectra (TEM-EDS, Tecnai F20, FEI Inc., Valley City, ND, USA) and scanning electron microscopic (SEM, Quanta 200S, FEI, Inc., Valley City, ND, USA) techniques. Thermogravimetric (TG) analyzer (TG, Netzsch, STA409C, Frankfurt, Germany) was applied to evaluate the thermal stability of the samples under nitrogen atmosphere.

### 4.3. Electrochemical Measurements

The cathode was composed of active materials, activated carbon and polyvinylidene fluoride (PVDF), with a mass ratio of 7:2:1, through mixing N-methyl-2-pyrrolidone (NMP) solvent to form slurry. After that, the slurry was pasted uniformly on a 316 L stainless-steel foil, subsequently heated at 100 °C overnight in oven and cut into Φ14 mm discs. The ZnSO_4_ solution (3 mol∙L^−1^) was applied as electrolyte, and zinc foil and glass-fiber film were applied as counter electrode and separator, respectively. The CR2025 coin batteries were assembled under normal air atmosphere conditions. The batteries were galvanostatically measured with various charge and discharge rates through an NEWARE testing instrument. The CHI660E electrochemical workstation is utilized to test the cyclic voltammetry (CV) and electrochemical impedance spectroscopy (EIS).

## 5. Conclusions

In summary, a facile and cost-effective V_2_O_3_@CD composite as a cathode for AZIBs was successfully fabricated via an evaporation-induced self-assembly process. The resultant composite reveals a unique structure in which V_2_O_3_ is homogeneously embedded in the macropores of bio-carbon with porous architectures. When V_2_O_3_@CD composites are employed as AZIB cathodes, the carbon skeleton can effectively enhance the conductivity and restrain the large volume change, guaranteeing the structural stability of the composites. As a consequence, the VOCD-3 composites exhibit the lowest R_ct_ of 187.7 Ω as well as a superior rate capability of 151 mAh g^−1^ at 3 A g^−1^ among the as-prepared V_2_O_3_@CD composites. In addition, the maximum capacity of 270 mAh g^−1^ at 1 A g^−1^ after 693 cycles is obtained in the VOCD-3 composites. It is believed that this study on a V_2_O_3_/biomass-derived carbon electrode could provide a new approach for the development of high-performance AZIBs using low-cost biomass as raw materials.

## Figures and Tables

**Table 1:** Comparison of the electrochemical performance of V_2_O_3_/CD composites in this work with other previously reported V-based cathode materials for AZIBs.

Materials	Method of Synthesis	Current Density (A g^−1^)	Specific Capacity (mAh g^−1^)	Capacity Retention	Cycle Number	Ref./Year
V_2_O_3_@carbonized dictyophora	evaporation-induced self-assembly technique	1	151.9	89.24%	1000	This work
V_2_O_3_@amorphous carbon	Calcination	1	116	90.7%	1600	[9]/2021
V_2_O_3_	Reduction method of boron	0.1	161	76.9%	100	[57]/2021
Carbon-coated NaVPO_4_F	CVD	0.1	87.4	94.5%	400	[58]/2021
V_2_O_x_@V_2_CT_x_	High-temperature etching and electrochemical active	1	87.3	81.6%	200	[59]/2020
V_2_O_5_ xerogel flakes	Hydrothermal	1	135	64%	200	[60]/2021
V_2_O_3_@Carbon Nanofibers	Electrospinning	0.2	120	80%	1000	[61]/2022
FeVO_4_•nH_2_O@rGO	Hydrothermal	1	92	43.8% ^a^	1000	[62]/2020

^a^ Values are estimated from the graphs.

**Table 2:** The dosage of each sample is summarized as follows.

Samples	Dictyophora	C_2_H_6_O_2_	CH_4_N_2_O	NH_4_VO_3_
VOCD-1	0.40 g	20 mL	0.75 g	0.55 g
VOCD-2	0.40 g	20 mL	1.50 g	1.10 g
VOCD-3	0.40 g	20 mL	3.00 g	2.20 g

## Data Availability

Not applicable.
